# Supplementary figures and images for: Cortical cell assemblies and their underlying connectivity: An in silico study
Source: PLoS Comput Biol. 2024 Mar 11;20(3):e1011891. doi: 10.1371/journal.pcbi.1011891 (PMC10927091; doi:10.1371/journal.pcbi.1011891)

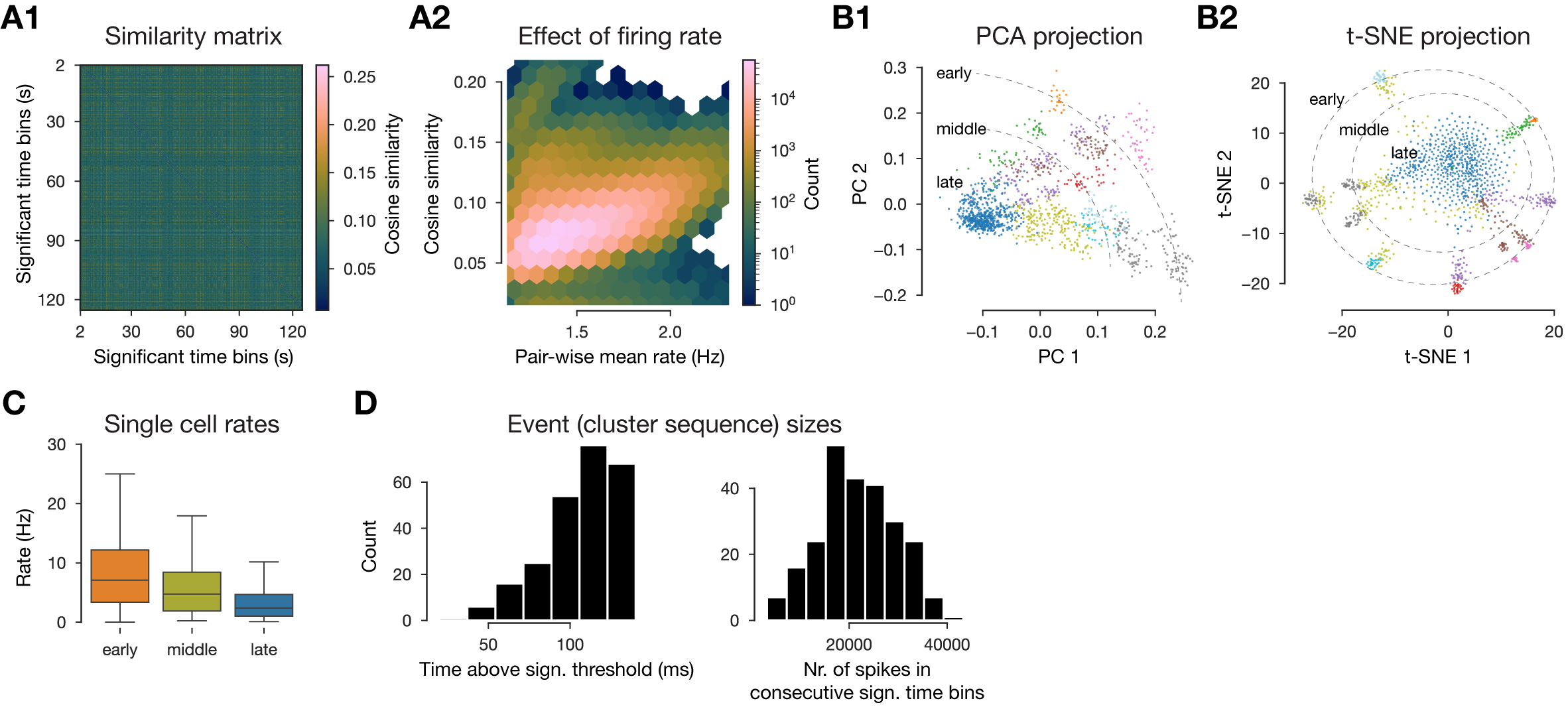

Supplement: S1 Fig — A1 Cosine similarity matrix (same as in Fig 3B1, but unsorted). A2: Joint distribution of pair-wise mean firing rate (within significant time bins) and cosine similarity. B1: 2D linear projection of mean centered and normalized spike matrix. B2: 2D nonlinear projection of spike matrix (using cosine distance). C: Single cell firing rates of neurons belonging to different assemblies. D: Distribution of stimulus evoked cluster sequence lengths (rows of matrices in Fig 3B2) and number of spikes in these significant time bins. (TIF) [file pcbi.1011891.s001.tif]

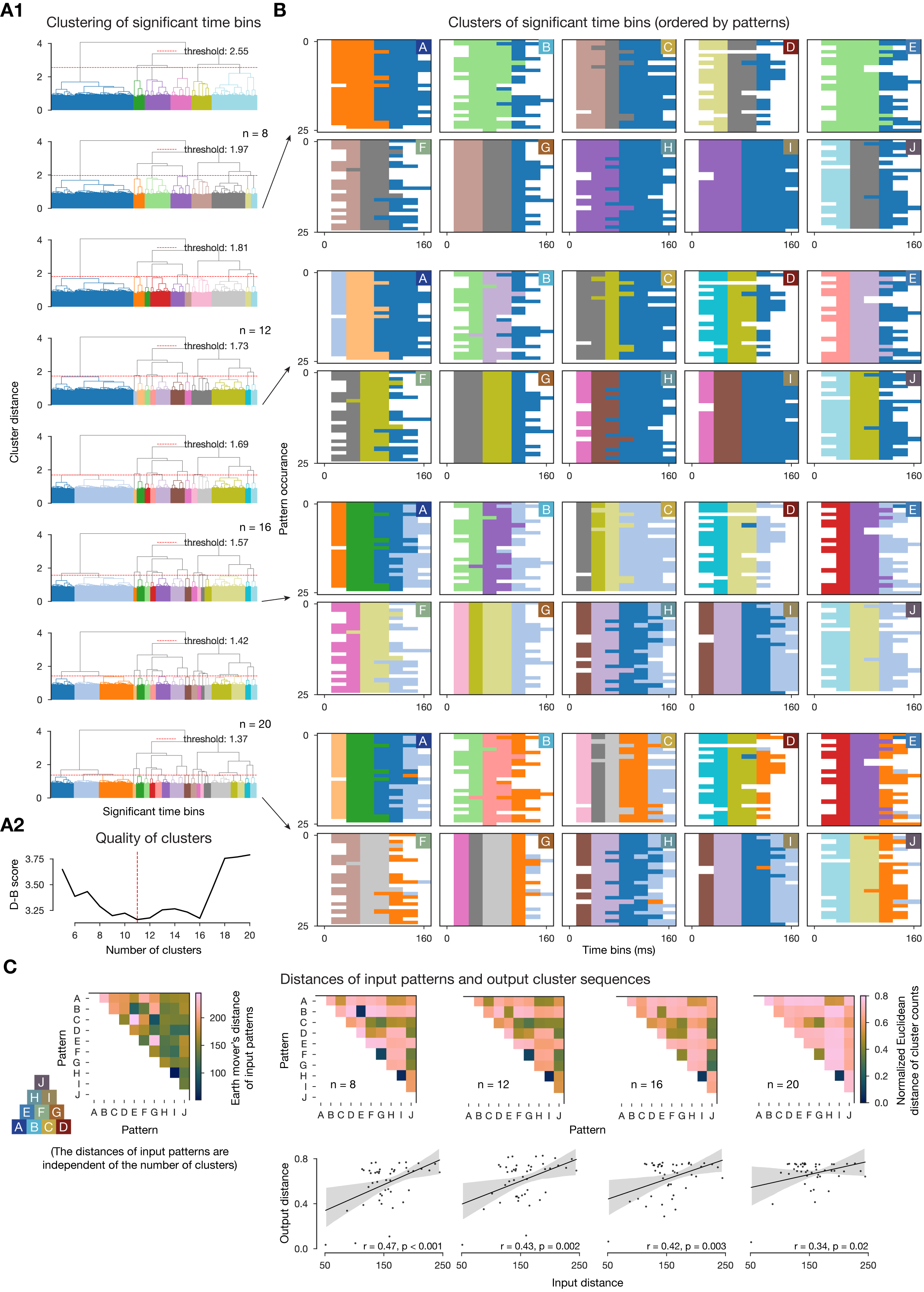

Supplement: S2 Fig — A1, B and C: As in Fig 3B and 3E but for different number of clusters (n). A2: Davis-Bouldin index [40] for different number of clusters. (TIF) [file pcbi.1011891.s002.tif]

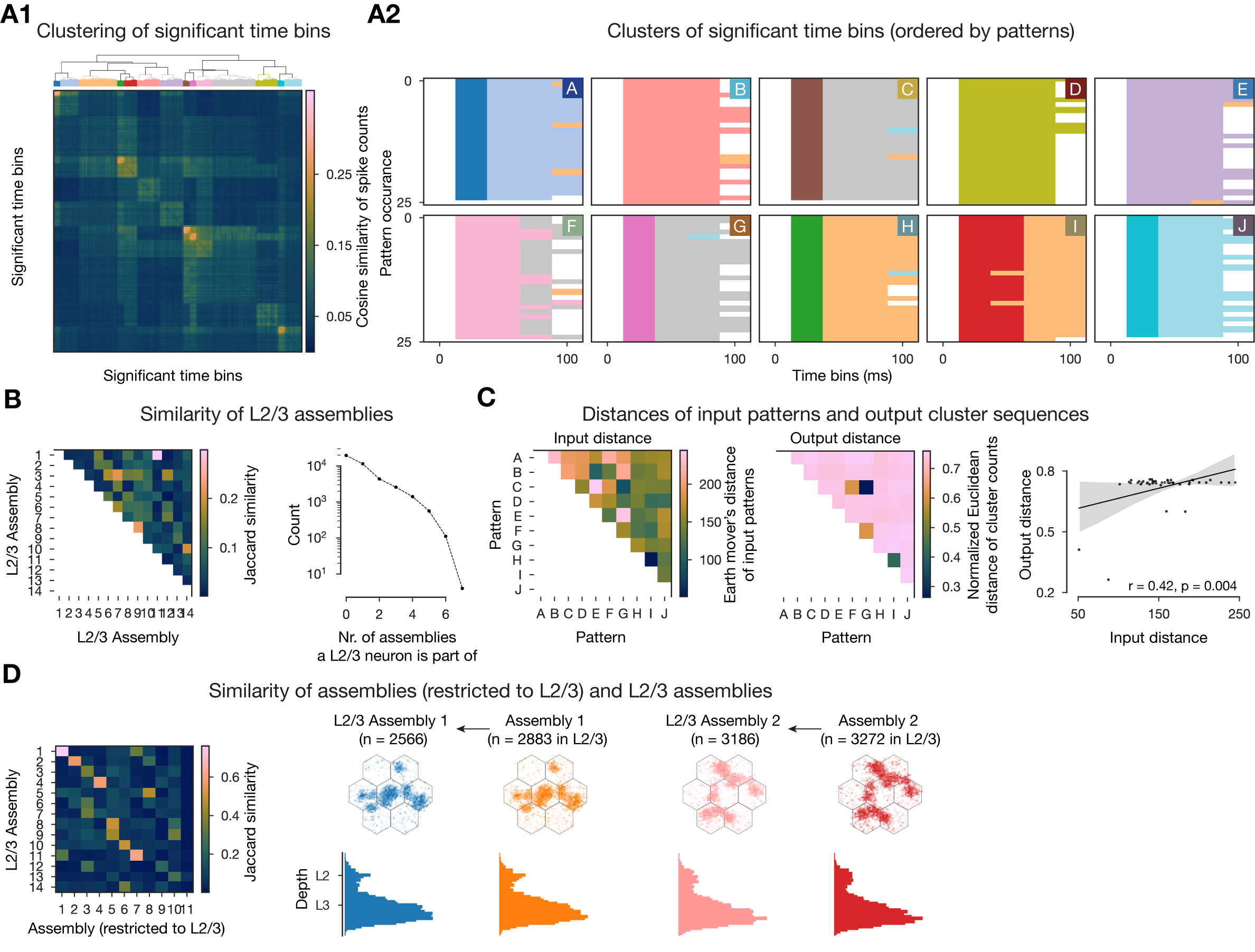

Supplement: S3 Fig — A-C: as in Fig 3B, 3D and 3E. d: Jaccard similarity of assemblies (detected across layers, but restricted to L2/3 here) and the ones detected in L2/3 on the left, and number and location: flatmap view on top, depth-profile below of exemplary pairs (pattern A and B responsive ones) of assemblies with high similarity to its right. (TIF) [file pcbi.1011891.s003.tif]

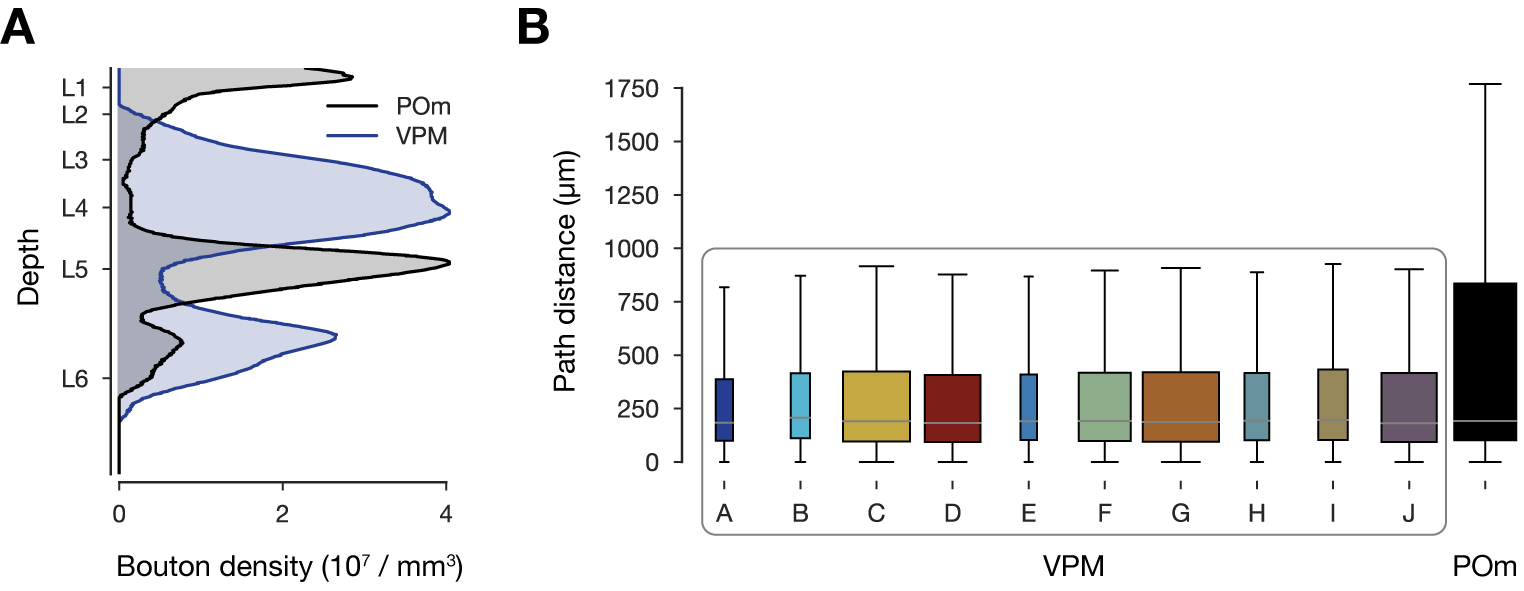

Supplement: S4 Fig — A: Density profile of VPM and POm synapses, digitized from Meyer et al. [41]. B: Synapse-to-soma path distances of different thalamocortical synapses on L5 neurons in an exemplary middle assembly (A8). Box widths represent the ratio of the number of synapses (e.g. most synapses on A8 L5 pyramidal cells are coming from pattern G, in line with the indegree based A8 membership probability on Fig 4B second). (TIF) [file pcbi.1011891.s004.tif]

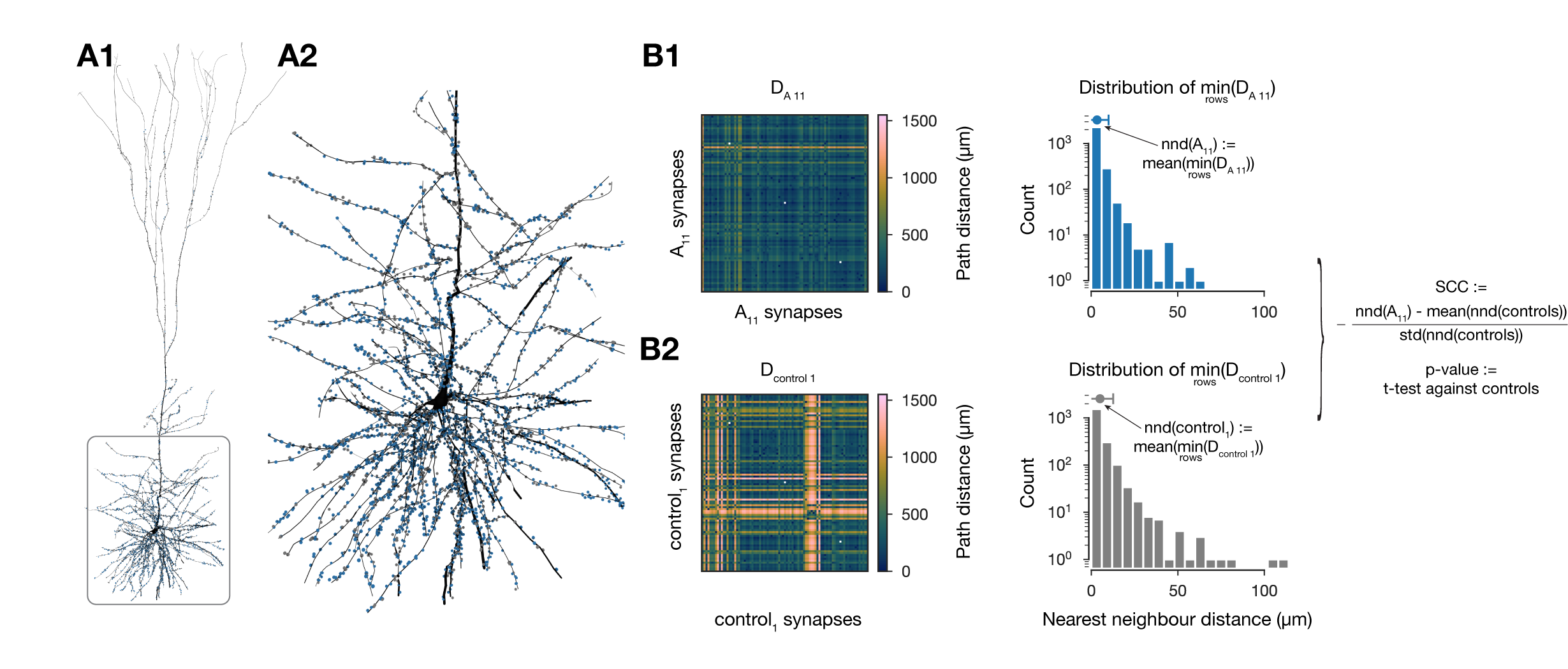

Supplement: S5 Fig — A1: Exemplary L5 pyramidal cell and all its afferent synapses from A11 (in blue) and from a control group (one out of the twenty) with the same number of presynaptic neurons (in gray). A2: Zoom in on A1. Soma, basal dendrites, and proximal apical dendrites are visible. Axon is not shown. The rendering was done with the BioExplorer package. B1: Distance matrix between all pairs of A11 synapses and distribution of nearest neighbour distances (minimum over the rows of the matrix) on its right (see Methods). B2: same as B1 but for the control group. The equations on the righmost part of the figure are motivated and explained in the Methods. (TIF) [file pcbi.1011891.s005.tif]

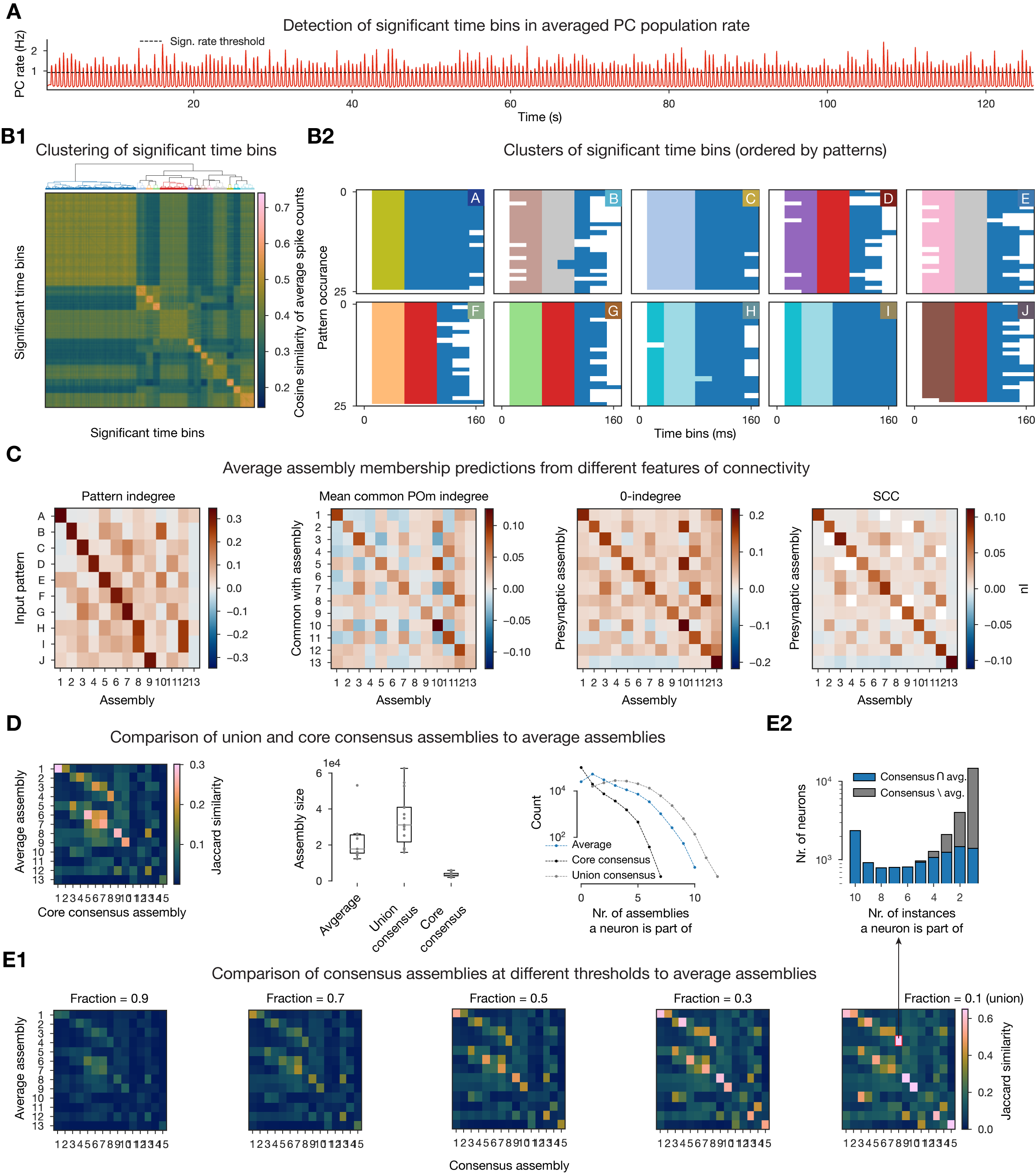

Supplement: S6 Fig — A, B: as in Fig 3A and 3B. C: as in Fig 6C. D: Left: Jaccard similarity of consensus assemblies and average assemblies. Middle: Number of neurons in conesensus assemblies’ union and core and average assemblies. Right: As middle, but number of neurons participating in given number of assemblies. E1: Jaccard similarity of consensus assemblies at different fraction thresholds (consensus assembly size grows to the right) and average assemblies. E2: Detailed comparison of the pair with the highest similarity in e1 at given number of assembly instances contained. (10 means that the consensus assembly neuron is part of 10/10 assembly instances, thus consensus assembly sizes grows to the right again.) Average \consensus is negligible (419 neurons) and is not shown. (TIF) [file pcbi.1011891.s006.tif]

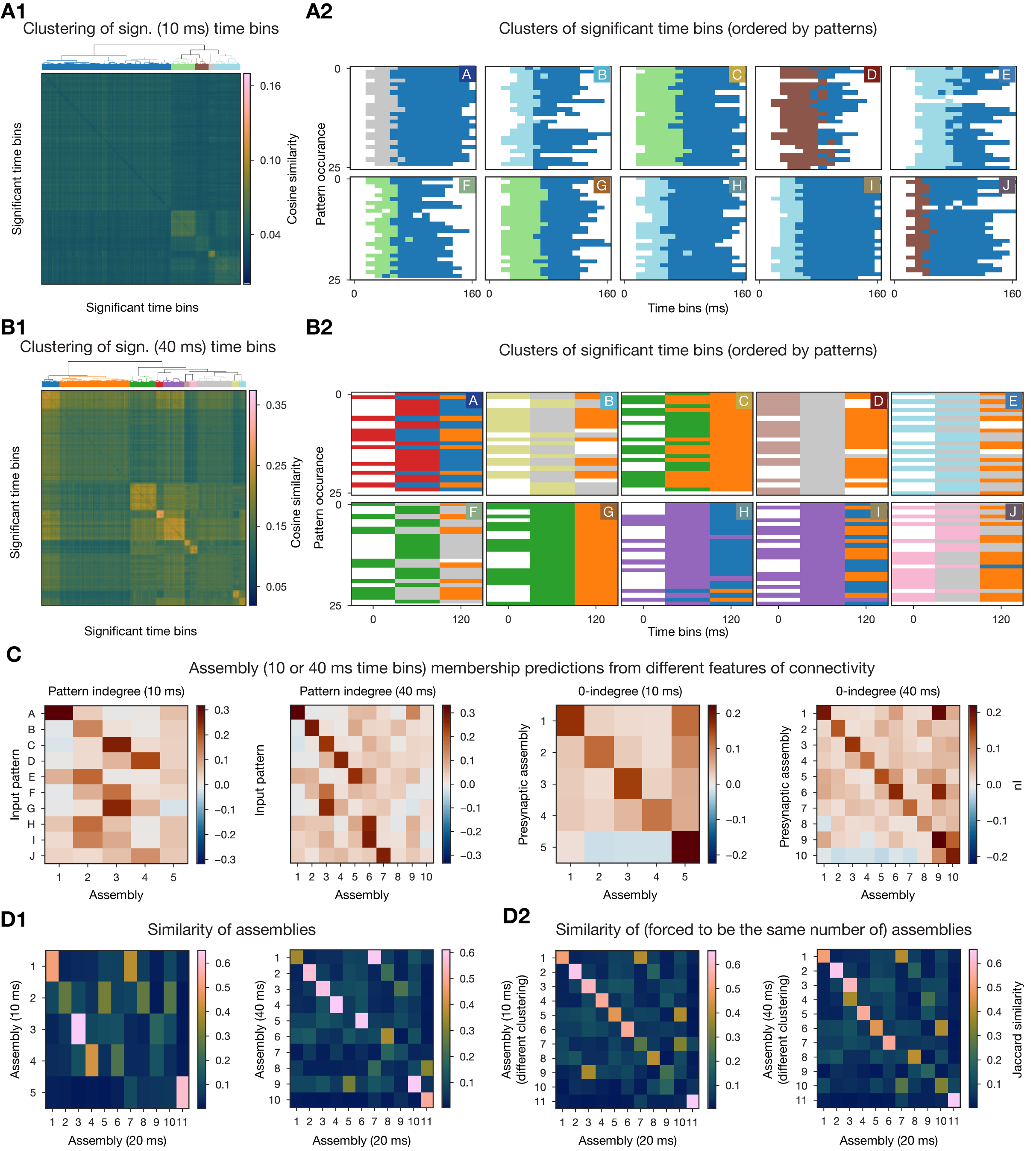

Supplement: S7 Fig — A-B: As in Fig 3B, but with different time bins. A: 10 ms, B: 40 ms (original: 20 20ms). C: As in Fig 4B and 4D. D1: Jaccard similiarity of assemblies detected this way and the original ones. D2: As D1, but the clustering tree cut at a different location (see S2 Fig) to result in 11 assemblies in the new cases as well. (TIF) [file pcbi.1011891.s007.tif]

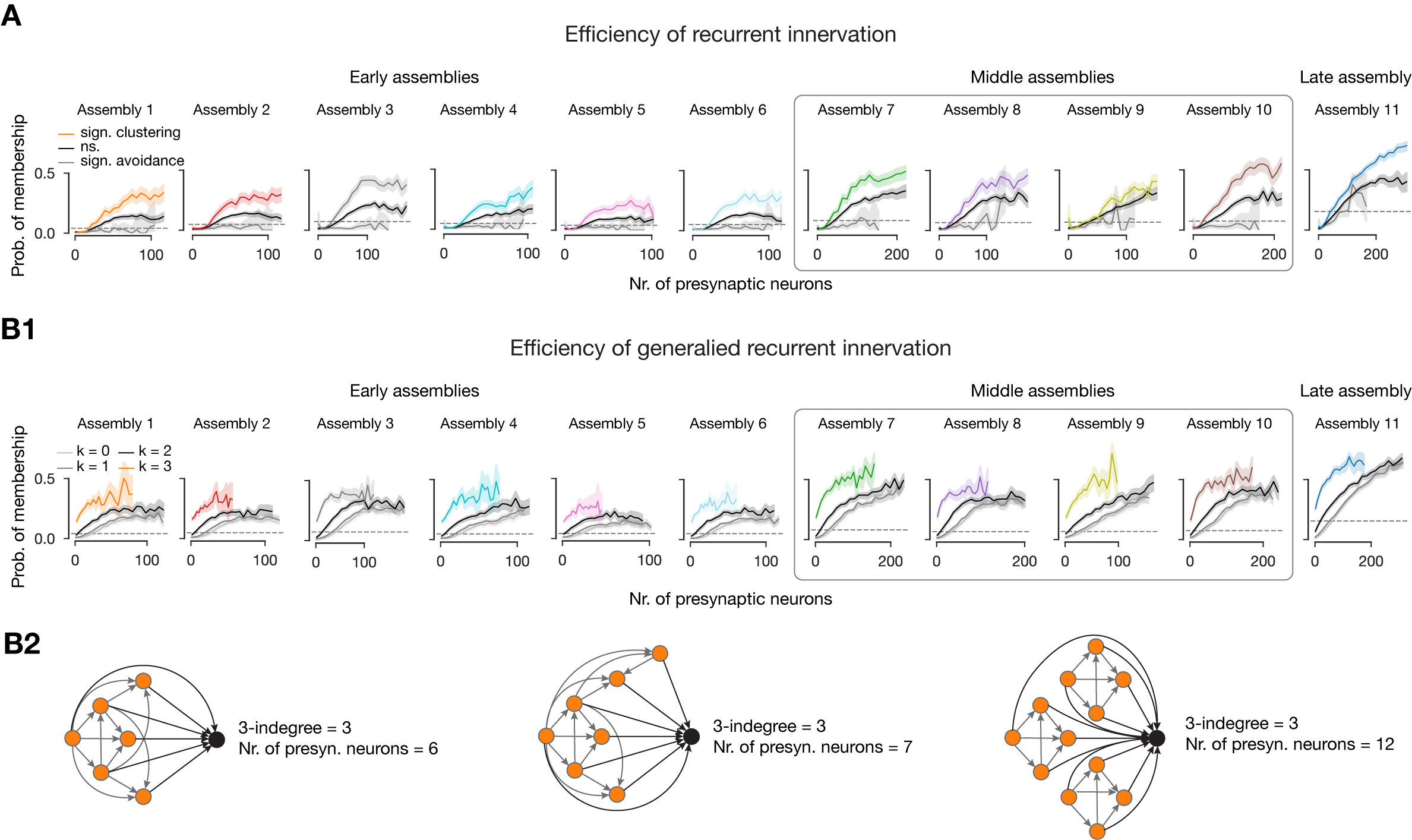

Supplement: S8 Fig — Probabilities of within assembly memberships for all assemblies. A: As in Fig 5B left. B1: Similar to Fig 4E left, but with different x-axis (presynaptic population size instead of simplex counts, see B2). B2: Illustration of the difference between k-indegree and presynaptic population size. (TIF) [file pcbi.1011891.s008.tif]

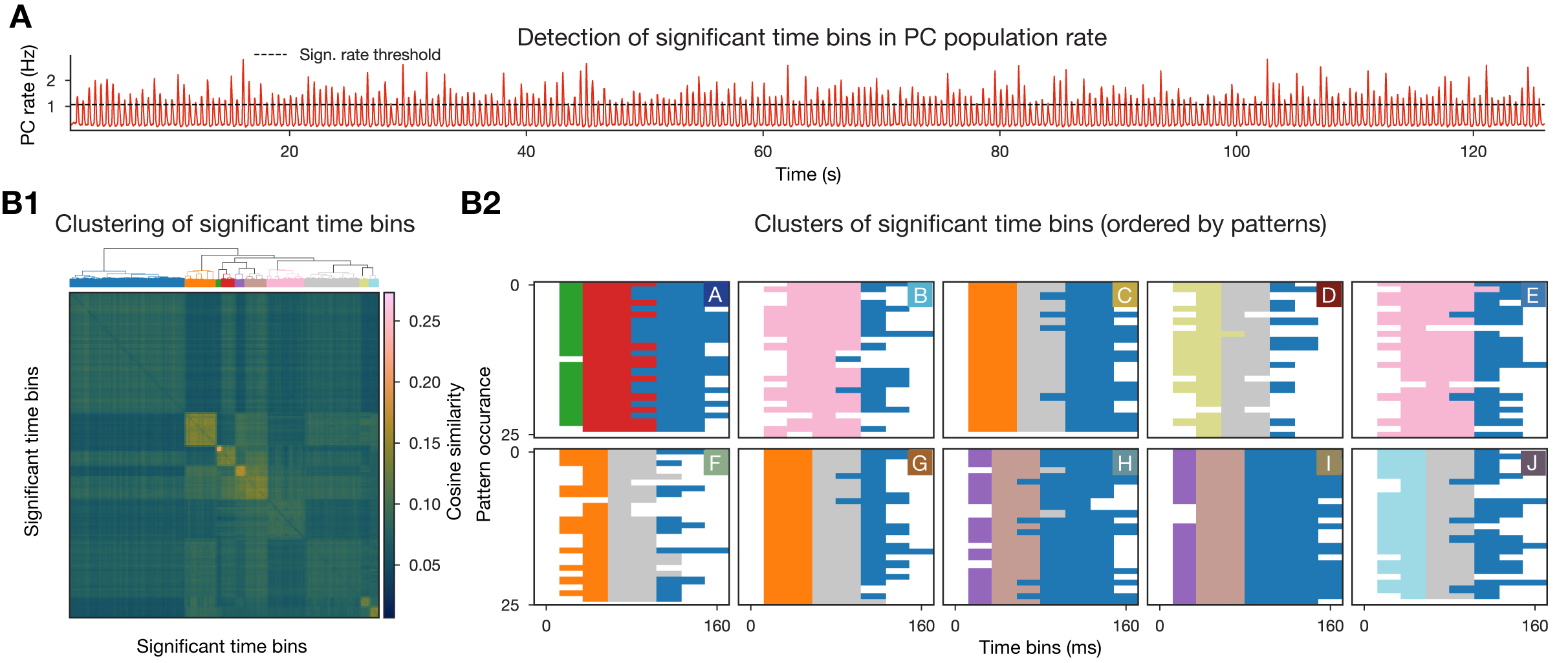

Supplement: S9 Fig — A-B As in Fig 3A and 3B, but the underlying simulation in this case does not have input from POm fibers. (TIF) [file pcbi.1011891.s009.tif]
