# Supplementary material for: Cortical cell assemblies and their underlying connectivity: An in silico study
Source: PLoS Comput Biol. 2024 Mar 11;20(3):e1011891. doi: 10.1371/journal.pcbi.1011891 (PMC10927091; doi:10.1371/journal.pcbi.1011891)
Supplement: Attachment — Submitted filename: Ecker_et_al_review_responses.pdf [file pcbi.1011891.s011.pdf]

Reviewer #1: # Summary and general comments

In this manuscript, Ecker, Santander et al conduct an in-silico study of a state-of-the art, biophysically detailed simulation of seven cortical columns of rat's primary somatosensory cortex. This model has been described and characterized in separate preprints (refs [35] and [36] in the text), as the reduced version (~200K neurons) of a larger model (4.2M neurons). The study is centered on the characterization of assembly-like, stimulus-evoked activity, and on the relation between assembly membership and circuit's anatomical structure. I find the work well written, original, technically sound, and quite remarkable in terms of biophysical realism. However it appears to me that most of the results are the direct outcome of the circuit's structural and dynamical properties. In summary, repeated patterns of activity are injected through thalamic afferents, activating consistently specific sets of neurons (early assemblies), delayed inputs facilitate activations in other sets of neurons (mid assemblies), finally recurrent connectivity causes further signal propagation across the whole population (late assembly). Factors such as calcium spikes and nonlinear interactions between clustered synapses also favor neural activations, when present, increasing transmission efficiency.

I find this work an interesting example of how a bottom-up approach can consistently and cohesively reveal large-scale circuit properties, offering more quantitative insights and predictions precluded to simpler models of pointwise neurons. Especially in quantifying more precisely how different circuit properties concur in generating evoked assembly-like activity. There are however some noteworthy limitations, that is important to mention.

Response*: We thank the reviewer for the accurate summary and hope that we were able to address their concerns with the following points*

First and foremost, the work focuses exclusively on assemblies directly evoked by injected stimuli, with no mentions of spontaneous activity. However a lot of interest is devoted to why and by which mechanisms assemblies emerge during spontaneous activity, and on the similarities between the spontaneous assemblies and those evoked by stimuli (e.g. refs [10,11,13,58] in main text). I think it should be explained why this aspect has not been investigated in the paper, despite its centrality in both experimental and modeling work.

Response: *We understand the importance of spontaneous assemblies and their link to evoked assemblies in the field and that we failed to acknowledge them in the previous version. We now explicitly mention spontaneous assemblies and work related to them in the introduction (lines 39-41; 70-72) and motivate our decision to focus on evoked assemblies (lines 73-74).*

Briefly, while we believe that our model is a suitable tool to study also spontaneous assemblies, it would require either the re-calibration of the spontaneous state (see Isbister et al., 2023, bioRxiv) for higher amplitude fluctuations in the total activity yielding a subset of the time bins to cluster. Without recalibration, spontaneous activity has low fluctuations in the total activity, i.e., nearly all time bins are significant. Thus, we would need to extend the current assembly detection methods in order to handle the large number of time bins and neurons present in our

simulations; which is computationally expensive. We therefore believe that this is outside the scope of this work.

Second, I find it curious that about 150 ms after stimulus onset the same, broadly distributed late assembly activates for every stimulus in the input set. This is an interesting model prediction I am not fully convinced about (it seems unjustified in terms of energy cost, for a patterns that seems quite uninformative). I also wonder how much of it is due to the presence of the broad, unspecific POm component that comes with every input stimulus. For example it would be interesting to see if it is still present in the absence of POm stimulus, and to what extent. Perhaps it should be better explained and justified why input stimuli have both a clustered and a more spread-out component.

Response: *We agree that such a non-specific assembly is unlikely to exist in vivo. We repeated the simulation campaign without the POm stimulus and found that this does not make the late assembly stimulus-specific (added as Supplementary Figure S9).*

We now present two hypotheses to explain the emergence of this phenomenon in the model in lines 452-463. A stimulus-specific late assembly may emerge through plasticity, or require interaction with other brain regions through feed-back connections and not be a phenomenon of the local circuitry alone. The second hypothesis is supported by the lower firing rate of the late assembly, as outlined in the text and in Supplementary Figure S1C.

Minor points

P0 - The manuscript does not have line numbers. I am surprised they are not a strict requirement, but even so please do add them in the future, as they would simplify the process of revising and editing.

Response: *We apologize for this oversight. We have added line numbers in this revision.*

Abstract - I find the mention of plasticity in the concluding sentence a bit confusing, since it is completely out of the scope of the work presented, and barely mentioned in the discussion.

Response: *We removed that sentence in this revision.*

P2, 4th paragraph. Typo on second line and question mark missing at end.

Response: *Typo fixed (line 56)*

P2, 5th paragraph. "tens of thousands" but it's more like 200K. Assemblies are described as "groups of neurons that fire together more than expected". Intuitively the definition makes sense to me, but I worry there might be a bit too much subjectivity in the meaning of "more than expected".

Response: *Changed to "over 200 thousand". We changed "than expected" to "than expected in shuffled controls" (line 64).*

P3, last paragraph of section 1. "Assemblies active after 50 ms are determined by recurrent connectivity". But later on the result is that "recurrent connectivity explains late assemblies". From Fig 3 B2 I would associate 50 ms with mid assemblies. (might be useful to offer a time range for mid and late assemblies)

Response: *We re-phrased this statement to be more qualitative about the impact of recurrent connectivity increasing and the impact of thalamic innervation decreasing the later an assembly is active (lines 85-87).*

P3, section 2.1 . Although the input is better described in the method section, I think you should describe the POm input as well. Initially I erroneously thought that VPM and POm stimuli belonged to separate sessions, rather than being delivered at the same time.

Response: *We made the simultaneous activation of POm and VPM fiber clearer in both the Results and Methods sections (lines 103-106 and 541; 560-561).*

Fig 3e I would show the y axis in the central panel as well.

Response: *We have updated the panel according to the suggestion.*

P5, section 2.1.1 Since the aim is not to build an ideal decoder, I don't think it can be stated that stimuli can "be distinguished better" in an analysis restricted to the L2/3 layer. I understand there is a better, one-to-one correspondence between detected assemblies and injected stimuli, but that would also be feasible in a full model with an optimal decoder, or with different assembly-selection criteria.

Response: *We changed the sentence according to the reviewer's suggestion (line 180-181).*

P7, section 2.2 In the definition of normalized mutual information, I would describe more clearly the distinction between having positive value, being zero, or having a negative value.

Response: *We completely re-wrote the section of the Results that explain this measure to be more intuitive and justify our additions to the standard mutual information. (Lines 197-206)*

P10 , end of 2.2.2 section, states that 0-indegree is equivalent to indegree. I think this should be clarified a bit earlier, given that the x-axis in Fig. 4D simply reads "indegree"

Response: *We updated the axis labels on Figure 4, moved the mentioning of 0-indegree being equivalent to indegree to the beginning of section 2.2.2 and kept using 0-indegree consistently afterwards.*

P14, "Our analysis support the idea that neuronal activity revolves around activation of assemblies". I find this statement a bit too strong: the analysis considers assemblies evoked in the model by specific stimuli chosen for this purpose. There is no comparison with alternative ideas and perspectives. The "idea that neuronal

activity revolves around activation of assemblies" seems to me the very starting point of this work.

Response: *We have deleted the offending sentence.*

P15, Discussion. I find the sentence "we are integrating the plasticity model of Chindemi et al" a bit too specific. It's surely important to indicate plasticity as possible future direction, but the conclusions could be a bit broader in scope.

Response: *That part of the discussion has been rewritten to also address other reviewer concerns. In the new version we better motivate and discuss potential future work studying assemblies in the context of plasticity. This discussion is now more general and no longer singles out the work of Chindemi et al. (lines 469-476)*

P17 , POm stimulus. I suggest to mention this in the results section as well. Also, it is not clear whether the POm fibers were randomly sampled only once for each VPM pattern and associated to it as frozen noise, or resampled independently at each stimulus presentation. The result section simply states "The stream consisted of repeated presentations of 10 different input patterns in random order", suggesting that they are sampled only once.

Response: *We made the frozen, non-stimulus specific nature of the POm part of the stimuli clearer in both the Results and Methods sections (lines 103-104 and 560; 563).*

Reviewer #2:

Assemblies of neurons firing together in the isocortex have been studied using in vivo recording methods, and their activity has been linked to different aspects of cortical coding. However, these in vivo methods lack information about structural connectivity, cell type identity, and subcellular synaptic specificity. This study uses a preexisting large, biophysically detailed model of rat somatosensory cortex to investigate the structural contributions to the formations of coactive neuronal assemblies. The authors quantify the effects of thalamic innervation, recurrent connectivity, subcellular targeting in determining membership of cell assemblies that occur at different times after stimulus presentation to the model. They find that the factors can be predictive, although the degree of contribution changes based on the time of assembly activity.

Overall, the study is quite interesting, well-designed, and generally well-presented. The use of a model where connectivity and neuronal identities are entirely known is a powerful approach to testing the influence of structural factors. This approach could also generate predictions for future in vivo experiments (especially those that are combined with post hoc connectivity measurements, like large-volume electron microscopy reconstruction). I only have a few suggestions to potentially improve the clarity of the manuscript or add information of interest to the community.

Response: *We thank the reviewer for the kind summary of our work.*

There are a few places where I felt there was a slight disconnect between the text and the figures. In the last paragraph of page 8, the authors discuss the notion of "k-indegree with respect to an assembly", and discuss results for 0-indegree before moving to 1-, 2-, and 3-indegree. The results for 0-indegree are presented in Figure 4D, but there is no specific mention of "0-indegree" (the authors just use the term "indegree" without a modifier), so it was unclear to me at first which results were specific to the 0-indegree analysis. I think it would be clearer if the "0-indegree" term was explicitly used in Fig. 4D. After this text, the authors then go on to say they will refer to 0-indegree as just "indegree" in later figures, but I think in Figure 4 it would better align with the text if "0-indegree" was explicitly used.

Response: *We addressed this together with a similar concern voiced by Reviewer #1 (we repeat that response here for easy finding). We updated the axis labels on Figure 4, moved the mentioning of 0-indegree being equivalent to indegree to the beginning of section 2.2.2 and kept using 0-indegree consistently afterwards.*

Also, for the results shown in Figure 6, the authors define a measure of "coreness" (discussed in the results and methods), but it seems as though this measure is not directly presented in Figure 6. Figure 6B has neurons grouped by "fraction of assemblies" instead, which seems to be similar to the "coreness" metric but not exactly the same. I think it would be clearer if a consistent measure were discussed and presented (or at least describe the differences between the measures).

Response: *We originally depicted in Figure 6 a measure that was related to, but slightly different from "coreness" as defined. We understand that this was needlessly confusing for the reader. We have updated Figure 6 to use coreness instead.*

Lastly, since cellular identity (as well as connectivity) is completely known in the model, I was curious if there were any cell-type specific results for assemblies or for the effect of innervating k-cliques. For example, are there k-cliques comprising particular combinations of cell types (types of inhibitory vs excitatory; types of excitatory cells within/across layers, etc.) that are more likely to lead to a cell joining an assembly than others? There is a fair amount of discussion of the layers in which the neurons belonging to different assemblies reside, but I think some additional analysis of cell type could be of great interest to readers interested in assigning circuit roles to particular types.

Response: *We appreciate the importance of understanding the roles of different classes of neurons and their interactions. However, in the context of an analysis of directed simplices, this becomes very challenging. The model contains 18 excitatory neuron types, each of them could be studied with respect to their roles as innervating or innervated neurons. Additionally, the effect can be considered for different simplex dimensions, and for different assemblies (most effects played out very differently for early, middle and late assemblies). This leads to a combinatorial explosion of different cases with many, possibly inter-dependent effects.*

To illustrate this, we considered the question of how 3-indegree from the late assembly affects morphological types (m-types) differently, see [Figure R1](#) in this document. Indeed, we can see that neurons of the L6 TPC:A,C and L5 TPC:A,B,C types were less affected in late assembly membership by having a low 3-indegree

than other types. However, this leads to knock-on questions: Is this a property specific to the late assembly? Is this a property of these types to be less affected by connectivity, or are there correlations with the other factors studied? (Such as thalamic inputs; also clustering could have a stronger effect due to larger dendrite sizes of thick-tufted classes). This further enhances the combinatorial explosion of things to study.*

That is why the additional dimension of neuron types is best studied not in an exploratory fashion, but with a specific hypothesis in mind. We believe this is out of scope for the present manuscript. We note that an interested reader could conduct analyses of the impact of neuron types using the data we made available.

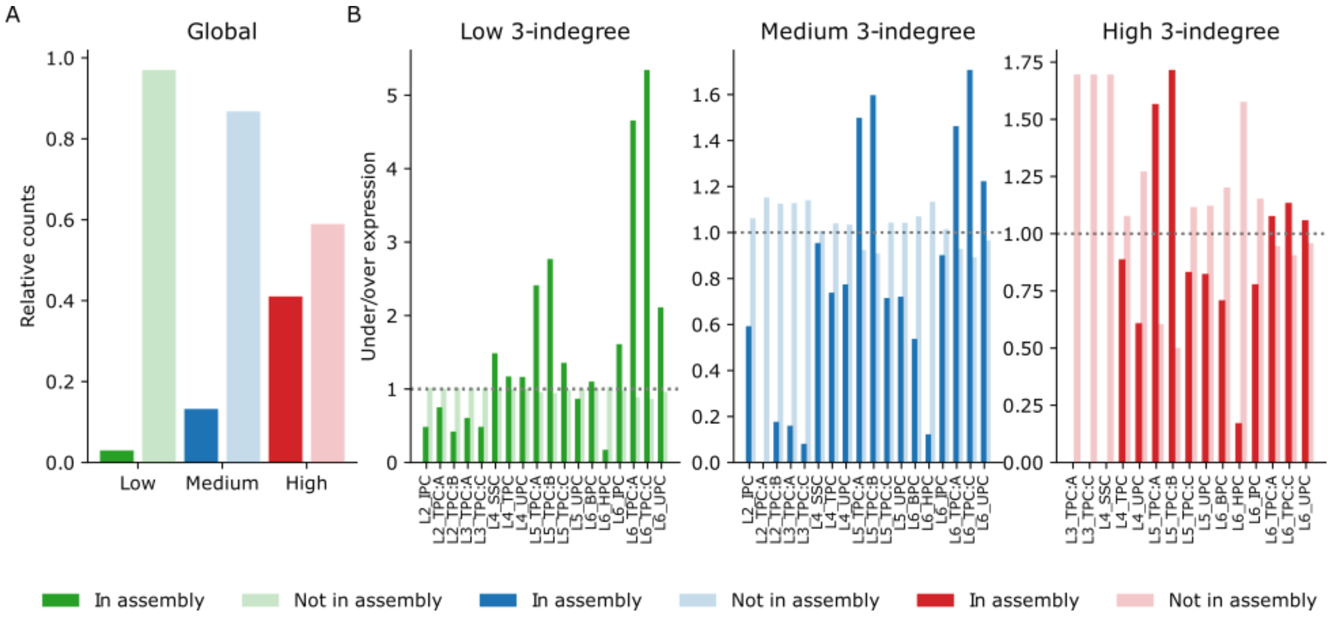

***Figure R1: Effect of mtype on assembly membership filtered by 3-indegree. A:** Global effect of 3-indegree from the late assembly in facilitating membership in the late assembly. Low (L) and High (H) 3-indegree from the late assembly represent 25% of the neurons with lowest and highest 3-indegree values respectively, the 50% remaining is classified as Medium (M) 3-indegree. **B:** Effect of mtype in assembly membership once the global effect depicted in panel A is normalized out. Left to right: three categories of Low (L), Medium (M) and High (H) 3-indegree from the late assembly. Each panel shows the probability that a neuron is of a given mtype, given that it has L/M/H 3-indegree and belongs or not to an assembly, normalized by the probability that a neuron is of that given mtype given that it has L/M/H 3-indegree irrespective of assembly membership. Values higher/lower than 1 show over/under expression of the neurons of that class.*

Overall, this is a strong and interesting study making good use of a large-scale, biophysically detailed model that should enable future directions of research.

***Response:** We once again thank the reviewer for the kind words.*

Reviewer 3:

This manuscript studied a very interesting topic: how are neural assemblies formed by simulating a large-scale in silico model. The in silico study can provide many

insights that cannot be discovered in current wet experiments. The study is comprehensive and timely. However, the comprehensive methods also bring up many questions for me, see blow.

Response: *We thank the reviewer for the positive words.*

Major:

- Can this study provide any experimentally testable prediction under current experimental methodology?

Response: *Yes, we believe that many of our predictions are testable, but we neglected to write about that in the discussion. We have added lines 443-449 to the discussion, where we outline our attempt to test our predictions in the MICrONS dataset, why it was not possible, and why we believe that it will be possible in the near future using additional EM datasets with co-registered activity data.*

- The study used so many “arbitrary” values to characterize the assembly. This begs the question if the conclusion depends on those choices? For example,

--although the authors cited Harris et al to validate the choice of 20 ms time bins, I am still curious would the conclusion change if this bin length changes.

Response: *We agree that demonstrating robustness of our methods with respect to parameter choices is important. We have therefore repeated our analyses with smaller and larger time bins and compared the results to the originally chosen time bins. We describe the results of this comparison in lines 412-419 and Supplementary Figure S7.*

--How did the authors justify the amplitude of inputs?

Response: *"We now outline the calibration of stimulus amplitudes in lines 556-560."*

- In main text/figure caption, it would be much better to intuitively and shortly explain the physical meaning (not only definition but what it can do) of many concepts and terms, such as input/output distance, clustering of significant time bins, k-simplices

Response: *We now provide a more intuitive explanation of the measure of normalized information (nI) in lines 197-206. We now provide short explanations of how to interpret the input and output distances in the caption of Figure 3. We provide an explanation of what the clustering method optimizes (minimization of total within-cluster variance) in lines 580-581. We explain k-simplices in the caption of Figure 4, where we first use them.*

- “We found that assemblies were activated in all stimulus repetitions and a series of two to three assemblies remained active for 110 \pm 30 ms”. It would be even nicer to show distribution of the assembly lifetime. Is it possible that the assembly is like the neural avalanche?

Response: *The relation of our work to the neural avalanches is indeed an interesting question. We explored the duration of assembly sequences and activated neuron counts more deeply (line 129-130 and Supplementary Figure S1D). Unlike for neural avalanches, we did not find distributions according to a power law. We believe that this is because we study exclusively stimulus-evoked assemblies (see also reply to Reviewer #1 above).*

- The assembly study ignores the inhibitory neuron. Why? Do they show similar behavior?

Response: *We now motivate our focus on the excitatory subpopulation only in the introduction (lines 76-77).*

- Fig. 7 shows that there are some areas of empty part, indicating there are other factors. What could be them?

together with:

- Together with the last question, I guess the dynamics is one of them or even a higher level factor than the connections that are studied by the authors. Dynamics is determined by many factors such as input (bottom up sensory inputs + top down inputs + noise), connectivity, single-neuron electrophysiology, synaptic dynamics, and E-I balance, etc. For example, below two papers contained results about how network dynamics affect assembly dynamics, form and its function.

Chen, Guozhang, and Pulin Gong. "[A spatiotemporal mechanism of visual attention: Superdiffusive motion and theta oscillations of neural population activity patterns.](https://www.science.org/doi/full/10.1126/sciadv.abl4995)" Science Advances 8.16 (2022): eabl4995.

Chen, Guozhang, and Pulin Gong. "[Computing by modulating spontaneous cortical activity patterns as a mechanism of active visual processing.](https://www.nature.com/articles/s41467-019-12918-8)" Nature communications 10.1 (2019): 4915.

Response: *We thank the reviewer for pointing out that we did not adequately explain this. Our structural measures indeed cannot fully explain assembly membership, as expected in a network with complex dynamics. We now explain this and outline relevant literature in lines 380-385*

- Sec. 4.1 shows that a single noise source is injected to all neurons. This is problematic because it gives rise to quite some correlation between neurons.

Response: *We are not injecting the same noise into all neurons. We apologize for the lack of clarity in that regard. We have updated the paragraph (line 489) to better explain that neurons receive noise with the similar statistics, but statistically independent.*

- Fig. 4A indicates that many neurons do not belong to even one assembly. Why? Due to the threshold in Fig. 3A?

Response: *We believe that the presence of neurons not belonging to any assembly is a consequence of our focus on evoked assemblies. We only analyze assemblies evoked by 10 stimuli; it is possible that different stimuli would engage previously unengaged neurons. We now explain this in lines 138-140.*

- The reader should at least get some intuitions about the method, for example, Ward's linkage, without reading another paper

Response: *We now provide an explanation of what the clustering method optimizes (minimization of total within-cluster variance) in lines 580-581.*

Minor:

- In Fig. 3 B2, what does the color represent?

Response: *Colors indicate assembly identity, which is used consistently throughout the manuscript (i.e., same color always refers to the same assembly). We now clarify this in the caption of Figure 3.*

- What are the corresponding controls in the first paragraph of 2.2.2?

Response: *We updated the sentence to spell out the controls used. (Lines 259-260)*

- It would be better to use a schematic diagram to illustrate what is VPM, Pom, recurrent connectivity in terms of neural anatomy.

Response: *We added small schematics to indicate which parts of Figure 4 present results related to VPM and POm innervation and which parts present results related to recurrent connectivity.*

- In methods, some of those distance metrics and many other values could be better explained by mathematical formulas.

Response: *We have added formulas for most distance metrics in the methods. (Lines 510; 518; 526; 531; 537).*

- "This begs the question: are we detecting different assemblies" Do the authors mean different assemblies in in vivo and in silico?

Response: *This is indeed what we meant. We re-phrased the sentence to clarify our intended meaning. (Lines 174-175.)*

- Fig. 4E and Fig. S7B1 and generlied -> generalized

- In Sec 4.4 forming and assembly -> forming an assembly; Note, that -> Note that; the the -> the

- Sec. 4.5, To asses -> To assess

Response: *We have fixed these typos.*

- What is unique benefit of normalized mutual information compared to mutual information? What extra knowledge can it provide?

Response: *While re-writing the explanation of normalized mutual information in response to criticism by Reviewer #1, we ensure to also explain the benefits of our modifications of the established mutual information measure. (Lines 197-206.)*

- The clustering of assembly activity in Fig. S1B1 is caused by their mean firing rate? The late assembly was not directly evoked by the input so its neurons' firing rate is lower?

Response: *We thank the reviewer for calling our attention to this. While finding the root cause of a clustering method, mean firing rate should be an affecting factor. Indeed, the late assembly has a low firing rate. We have added this finding in Supplementary Figure S1C and further explain it in the Discussion (lines 453-456.)*
