# Supplementary material for: Cortical cell assemblies and their underlying connectivity: An in silico study
Source: PLoS Comput Biol. 2024 Mar 11;20(3):e1011891. doi: 10.1371/journal.pcbi.1011891 (PMC10927091; doi:10.1371/journal.pcbi.1011891)
Supplement: Attachment — Submitted filename: review.docx [file pcbi.1011891.s010.docx]

This manuscript studied a very interesting topic: how are neural assemblies formed by simulating a large-scale in silico model. The in silico study can provide many insights that cannot be discovered in current wet experiments. The study is comprehensive and timely. However, the comprehensive methods also bring up many questions for me, see blow.

Major:

- Can this study provide any experimentally testable prediction under current experimental methodology?

- The study used so many “arbitrary” values to characterize the assembly. This begs the question if the conclusion depends on those choices? For example,

--although the authors cited Harris et al to validate the choice of 20 ms time bins, I am still curious would the conclusion change if this bin length changes.

--How did the authors justify the amplitude of inputs?

- In main text/figure caption, it would be much better to intuitively and shortly explain the physical meaning (not only definition but what it can do) of many concepts and terms, such as input/output distance, clustering of significant time bins, k-simplices

- “We found that assemblies were activated in all stimulus repetitions and a series of two to three

assemblies remained active for 110 \pm 30 ms”. It would be even nicer to show distribution of the assembly lifetime. Is it possible that the assembly is like the neural avalanche?

- The assembly study ignores the inhibitory neuron. Why? Do they show similar behavior?

- Fig. 7 shows that there are some areas of empty part, indicating there are other factors. What could be them?

- Together with the last question, I guess the dynamics is one of them or even a higher level factor than the connections that are studied by the authors. Dynamics is determined by many factors such as input (bottom up sensory inputs + top down inputs + noise), connectivity, single-neuron electrophysiology, synaptic dynamics, and E-I balance, etc. For example, below two papers contained results about how network dynamics affect assembly dynamics, form and its function.

Chen, Guozhang, and Pulin Gong. "[A spatiotemporal mechanism of visual attention: Superdiffusive motion and theta oscillations of neural population activity patterns.](https://www.science.org/doi/full/10.1126/sciadv.abl4995)" Science Advances 8.16 (2022): eabl4995.

Chen, Guozhang, and Pulin Gong. "[Computing by modulating spontaneous cortical activity patterns as a mechanism of active visual processing.](https://www.nature.com/articles/s41467-019-12918-8)" Nature communications 10.1 (2019): 4915.

- Sec. 4.1 shows that a single noise source is injected to all neurons. This is problematic because it gives rise to quite some correlation between neurons.

- Fig. 4A indicates that many neurons do not belong to even one assembly. Why? Due to the threshold in Fig. 3A?

- The reader should at least get some intuitions about the method, for example, Ward’s linkage, without reading another paper

Minor:

- In Fig. 3 B2, what does the color represent?

- What are the corresponding controls in the first paragraph of 2.2.2?

- It would be better to use a schematic diagram to illustrate what is VPM, Pom, recurrent connectivity in terms of neural anatomy.

- In methods, some of those distance metrics and many other values could be better explained by mathematical formulas.

- “This begs the question: are we detecting different assemblies” Do the authors mean different assemblies in in vivo and in silico?

- Fig. 4E and Fig. S7B1 and generlied -🡪 generalized

- In Sec 4.4 forming and assembly 🡪 forming an assembly; Note, that 🡪 Note that; the the 🡪 the

- Sec. 4.5, To asses 🡪 To assess

- What is unique benefit of normalized mutual information compared to mutual information? What extra knowledge can it provide?

- The clustering of assembly activity in Fig. S1B1 is caused by their mean firing rate? The late assembly was not directly evoked by the input so its neurons’ firing rate is lower?
